# Germline and somatic mutations in cortical malformations: Molecular defects in Argentinean patients with neuronal migration disorders

**DOI:** 10.1371/journal.pone.0185103

**Published:** 2017-09-27

**Authors:** Dolores González-Morón, Sebastián Vishnopolska, Damián Consalvo, Nancy Medina, Marcelo Marti, Marta Córdoba, Cecilia Vazquez-Dusefante, Santiago Claverie, Sergio Alejandro Rodríguez-Quiroga, Patricia Vega, Walter Silva, Silvia Kochen, Marcelo Andrés Kauffman

**Affiliations:** 1 Consultorio y laboratorio de Neurogenética, Centro Universitario de Neurología “José María Ramos Mejía” y División Neurología, Hospital JM Ramos Mejía, Facultad de Medicina, UBA, Buenos Aires, Argentina; 2 IBCN “Eduardo de Robertis”, Facultad de Medicina, UBA-CONICET, Buenos Aires, Argentina; 3 Universidad de Buenos Aires, Facultad de Ciencias Exactas y Naturales, Departamento de Química Biológica, Buenos Aires, Argentina; 4 CONICET, Universidad de Buenos Aires, Instituto de Química Biológica de la Facultad de Ciencias Exactas y Naturales (IQUIBICEN), Buenos Aires, Argentina; 5 Centro Universitario de Neurología “José María Ramos Mejía” y División Neurología, Hospital JM Ramos Mejía, Facultad de Medicina, UBA, Buenos Aires, Argentina; 6 Sección Neurofisiología y Epilepsia, Consultorio de Epilepsias Refractarias, Servicio de Neurología Infantil, Hospital Italiano de Buenos Aires, Buenos Aires, Argentina; 7 Centro de Epilepsia, División Neurología, Hospital JM Ramos Mejía, Facultad de Medicina, UBA, Buenos Aires, Argentina; 8 Programa de Medicina de Precisión y Genómica Clínica, Instituto de Investigaciones en Medicina Traslacional, Facultad de Ciencias Biomédicas, Universidad Austral-CONICET, Derqui, Argentina; NIDCR/NIH, UNITED STATES

## Abstract

Neuronal migration disorders are a clinically and genetically heterogeneous group of malformations of cortical development, frequently responsible for severe disability. Despite the increasing knowledge of the molecular mechanisms underlying this group of diseases, their genetic diagnosis remains unattainable in a high proportion of cases. Here, we present the results of 38 patients with lissencephaly, periventricular heterotopia and subcortical band heterotopia from Argentina. We performed Sanger and Next Generation Sequencing (NGS) of *DCX*, *FLNA* and *ARX* and searched for copy number variations by MLPA in *PAFAH1B1*, *DCX*, *POMT1*, and *POMGNT1*. Additionally, somatic mosaicism at 5% or higher was investigated by means of targeted high coverage NGS of *DCX*, *ARX*, and *PAFAH1B1*. Our approach had a diagnostic yield of 36%. Pathogenic or likely pathogenic variants were identified in 14 patients, including 10 germline (five novel) and 4 somatic mutations in *FLNA*, *DCX*, *ARX* and *PAFAH1B1* genes. This study represents the largest series of patients comprehensively characterized in our population. Our findings reinforce the importance of somatic mutations in the pathophysiology and diagnosis of neuronal migration disorders and contribute to expand their phenotype-genotype correlations.

## Introduction

Malformations of cortical development (MCD) are a major cause of intellectual disability and severe epilepsy. Among them, neuronal migration disorders (NMD), resulting from a disruption in the normal movement of neurons from their original birth site to their final location at early brain developmental phases [1] can be considered a sub-group with syndromic defining imaging features including periventricular nodular heterotopia (PNH), subcortical band heterotopia (SBH), also called double cortex syndrome (DC), and lissencephaly [2].

Despite recent increase in our knowledge of NMD genetics, the identification of disease-causing mutations in individual patients remains a challenge on clinical grounds, even in the Next Generation Sequencing (NGS) era. A high proportion of cases from NMD cohorts persists as genetically undefined [3]. Moreover, somatic mosaicism could be the mechanism underlying the aetiology of a third of these patients [4]. Therefore, their molecular diagnosis should be addressed to look not only for germline, but for somatic variants as well.

We aimed to test the utility, feasibility and diagnostic yield of implementing a comprehensive molecular diagnostic strategy for NMD, by assessing the presence of germline mutations, copy number variants and somatic mutations in six of the main NMD causing genes in a cohort of MCD patients from an academic centre in a developing country.

## Materials and methods

### Patients

As part of our on-going MCD registry (beginning in 2007) patients with NMD were referred to our Neurogenetic Clinic settled in a tertiary academic centre in Argentina. Among them, patients with MRI features characteristic of periventricular nodular heterotopia (PNH), subcortical band heterotopia (SBH) and lissencephaly (LIS) syndromes were selected. Detailed information regarding family history (pedigree analysis), pre- and perinatal events, epilepsy, psychomotor development, cognitive function, associated systemic malformations, and neurological examination was collected.

### Neuroimages

Magnetic resonance images (MRI) of 1.5 T were available for all patients and were analyzed by the same specialist in neuroimages for the number and localization of nodular heterotopias in PNH, the extension, localization and thickness of the band in SBH (as previously described in [5]) and gradient of lissencephaly. The gradient comprises the severity of LIS along the anterior–posterior axis of the brain, which could be more severe in the frontal region (anterior greater than posterior, or a >p), equal (anterior similar to posterior, or a = p), or more severe in the parieto-occipital region (posterior greater than anterior, or p >a). Similarly, the presence of other anomalies affecting CNS was reviewed (i.e. agenesis of the corpus callosum). Patients with PNH were further classified according to the radiological findings in *typical PNH* and *atypical PNH*. The criteria for *typical PNH* included bilaterally grey matter lining the lateral ventricles with otherwise normal appearing white and cortical grey matter. Asymmetric heterotopia and associated findings of cerebellar hypoplasia, enlarged cisterna magna and thinning or agenesis of the corpus callosum were also accepted in *typical PNH* patients. Patients with subcortical heterotopia, disorders of the overlying cortex and nodules localized predominantly along the atria and temporal horns of the lateral ventricles were considered *atypical PNH*.

### Diagnostic approach. Genetic assays

**Fig 1:**
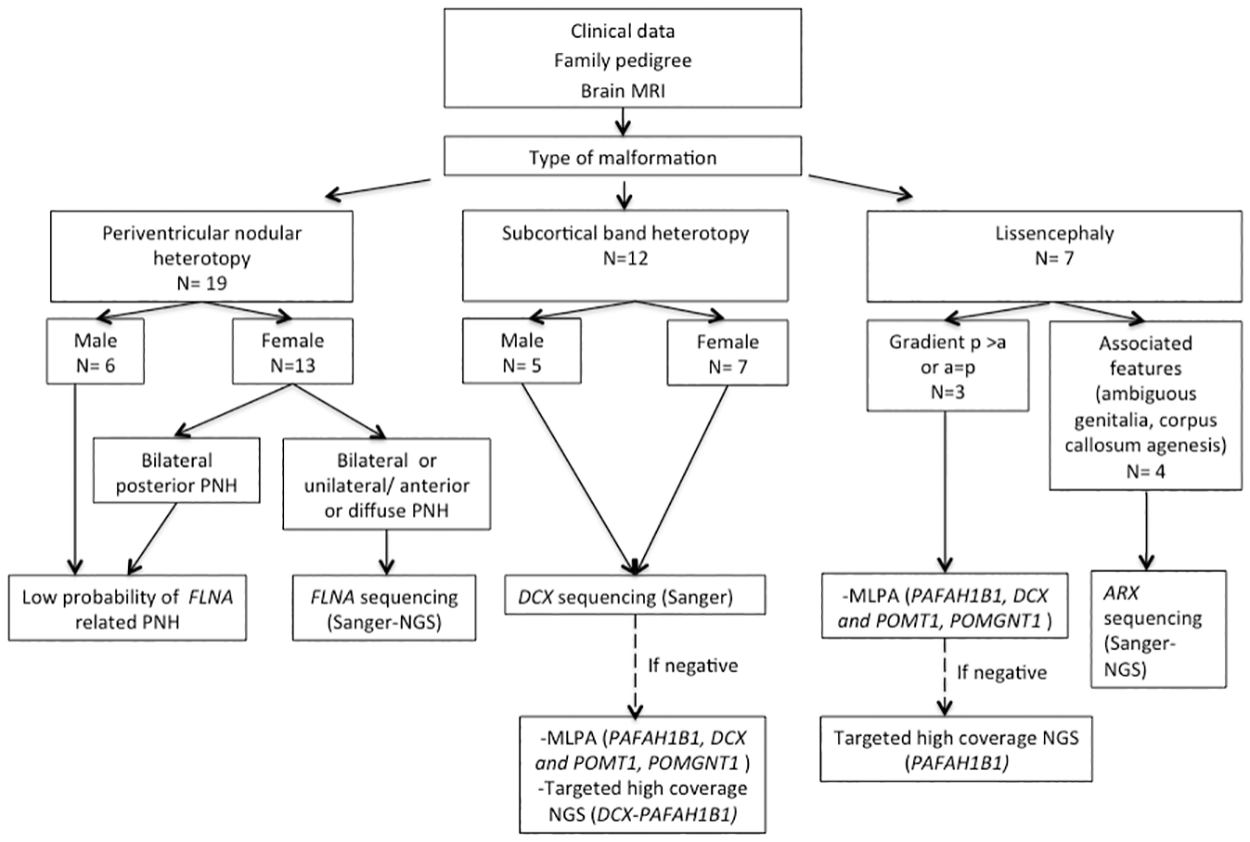
Molecular workflow. General scheme describing the workflow of molecular tests performed for each type of malformation.

We performed Sanger sequencing of *DCX*, *ARX*, and *FLNA* in patients with SBH, lissencephaly with ambiguous genitalia and/or corpus callosum agenesis and typical PNH, respectively. Patients with atypical PNH, particularly those with bilateral posterior PNH were excluded from the *FLNA* mutational analysis as, according to previous evidence, they were presumed to be distinct entities (*FLNA* negative) [6, 7]. In addition, we applied multiplex ligation-dependent probe amplification (MLPA) to detect copy number variants (CNV) disrupting *PAFAH1B1*, *DCX* and *POMT1*, *POMGNT1* in all individuals with agyria-pachygyria spectrum (lissencephaly and SBH). Those patients with SBH and a mutation involving *DCX* detected during *DCX* sequencing were excluded from CNV analysis. Finally, we applied targeted high-coverage next generation sequencing for the detection of somatic mutations in *DCX*, *PAFAH1B1*, and *ARX* when previous assays were negative (Fig 1).

#### Sanger and NGS of NMD causing genes

All coding regions and exon-intron boundaries of *DCX* and *ARX* genes were amplified by polymerase chain reaction (primers and conditions are available upon request) and sequenced through Sanger sequencing by capillary electrophoresis (ABI BigDye terminator).

Depending on the date of inclusion of each patient, the coding sequence of *FLNA* was interrogated by means of Sanger sequencing, 454-based NGS or Illumina MiSeq-based NGS. In both NGS assays, the first step of *FLNA exonic enrichment* was based on a long-range PCR (LR-PCR) approach as previously described by us for mitochondrial disorders [8]

#### CNV detection. MLPA

MLPA test was performed with the P061 kit, lot B1 (MRC HOLLAND), following the procedure specified by the developer (http://www.mrc-holland.com). Case-control samples were processed under the same conditions and in the same experiment. The PCR products were quantified by capillary electrophoresis on an ABI 31xl analyzer. Electropherograms were analyzed using the software Coffalyser V 1.0 (MRC Holland).

#### Somatic mutations. Targeted high coverage NGS

Exons of the *DCX*, *PAFAH1B1* and *ARX* genes were amplified by LR-PCR using a Q5^®^ High-Fidelity DNA Polymerase (New England BioLabs Inc.). All amplified fragments for the same patient were mixed in the same tube and purified of the PCR reagents. Each pool was prepared for sequencing following the “Nextera XT DNA Sample Preparation Workflow” protocol and sequenced on Illumina MiSeq for high depth sequencing (> 1000X). The resulting FASTQ files were filtered with PRINSEQ (mean quality score > 10) and mapped to the human reference genome (GRCh37.75) with BWA [9]. The resulting SAM was sorted and converted to BAM with PICCARD and the alignment was recalibrated following the Broad Institute recommended best practices [10, 11]Variants were called using Haplotype Caller (GATK) [12] and Platypus[13]. Structural variants (big deletion or insertions among the amplified fragments) were called with Delly2[14]. The resulting VCF files were annotated with structural information with SnpEff [15] to detect variations with moderate or high impact. Allelic frequency was calculated considering the number of individual reads evidencing the mutations in relation to the total number of sequences covering that region. All pathogenic variants detected by NGS were validated. Variants for which the alternate-allele read frequency was 40% or lower were evaluated for mosaicism by subcloning. Original DNA was re-amplified by means of a polymerase chain reaction (PCR) assay, subcloned into a linearized vector (pGEM(R)-T Easy Vector System II PROMEGA) and transformed into JM109 Competent Cells (Promega). Multiple individual transformants were re-isolated, and Sanger sequencing was performed to confirm the presence or absence of the predicted variant and to quantify the degree of mosaicism.

Reference sequences used: *FLNA* (NM_001110556), *DCX* (NM_178152.1), *ARX* (NM_1390582), *PAFAH1B1* (NM_000430)

### Statements

This study was approved by our Institutional Ethics Committee (CODEI, Buenos Aires, Argentina). All patients and parents provided written informed consent for genetic analyses and use of their anonymized data. All experiments and methods were carried out in accordance with the relevant guidelines and regulations of CODEI. All clinical investigations have been conducted according to the principles expressed in the Declaration of Helsinki.

## Results

From a cohort of 70 MCD patients, we selected 38 patients with NMD. The most frequent type of malformation was PNH (N = 19), followed by SBH (N = 12) and lissencephaly (N = 7) (S1 Table). A conclusive genetic diagnosis was achieved in 14 patients. All variants reached ACMG [16] criteria to be classified as pathogenic or likely pathogenic (for allele frequency and in silico analysis see S2 and S3 Tables). Thus, the diagnostic yield of our comprehensive approach was 36%. Clinical and genetic findings are summarized in Tables 1, 2 and 3. Further details and illustrative cases are presented below.

**Table 1:** Phenotypic characteristics of individuals with a molecular diagnosis.

PARTICIPANT NUMBER	SEX	PHENOTYPE		ASSOCIATED SNC MALFORMATION	SYSTEMIC MANIFESTATIONS	INTELLECTUAL DISABILITY	EPILEPSY	FAMILY HISTORY
		CORTICAL MALFORMATION	DISTRIBUTION					
MDC1002	F	PNH	Bilateral. Symmetric	Vermis hypoplasia	Personality disorder	No	Yes	Sporadic
MDC1005	F	PNH	Bilateral. Symmetric	Right WM changes	Cardiac. VSD	No	Yes	Sporadic
MDC1019	F	PNH	Bilateral. Symmetric	No	No	No	Yes	Spontaneous abortions
MDC1020	F	PNH (Isolated single nodule)	Unilateral. Lateral ventricle, frontal horn	No	No	No	No	Spontaneous abortions
MDC1080	F	SBH	Diffuse A = P	No	No	Yes	Yes	Sporadic
MDC1045	F	SBH	Diffuse A > P	No	No	Yes	Yes	Lissencephaly
MDC1063	F	SBH	Diffuse A = P	No	No	Yes	Yes	Sporadic
MDC1075	M	Lissencephaly	Diffuse P>A	No	No	Yes	Yes	Sporadic
MDC1009	M	Lissencephaly	Diffuse A = P	CC and septum pellucidum agenesis	diarrhoea, acidosis, hypothermia, ambiguous genitalia (XLAG)	Yes	Yes	Sporadic
MDC1039	M	Lissencephaly	Diffuse A = P	CC agenesis	ambiguous genitalia (XLAG)	Yes	Yes	Sporadic
MDC1092	M	SBH	Diffuse A = P	No	No	Yes	Yes	Sporadic
MDC1093	F	SBH	Diffuse A>P	No	No	No	Yes	Sporadic
MDC1034	M	SBH	Diffuse A = P	No	No	Yes	Yes	Sporadic
MDC1070	F	SBH	Diffuse P>A	No	No	Yes	Yes	Sporadic

PNH Periventricular Nodular Heterotopia; WM white matter; CC Corpus Callosum; HBS Subcortical Band Heterotopia; VSD Ventricular septal defect; A anterior; P posterior, VSD Ventricular septal defect, XLAG X-linked lissencephaly with abnormal genitalia

**Table 2:** Details of germline mutations.

PARTICIPANT NUMBER	CORTICAL MALFORMATION	GENOTYPE			
		GENE	MUTATION/ CNV	PREVIOUSLY REPORTED	VARIANT CLASSIFICATION (ACMG CONSENSUS)
MDC1002	PNH	FLNA	NM_001110556.1: c.4543C>T; p.R1515X	Yes ^1^	Pathogenic
MDC1005	PNH	FLNA	NM_001110556.1: c.2193C>G; p.Y731X	Yes ^2^	Pathogenic
MDC1019	PNH	FLNA	NM_001110556.1: c.4159G>A; p.G1387S	No	Likely pathogenic
MDC1020	PNH (Isolated single nodule)	FLNA	NM_001110556.1: c.4159G>A; p.G1387S	No	Likely pathogenic
MDC1080	SBH	DCX	NM_178152.1: c.305G>C; p. R102P	No	Likely pathogenic
MDC1045	SBH	DCX	NM_178152.1: c.640T>C; p.I214L	Yes ^3^	Pathogenic
MDC1063	SBH	DCX	NM_178152.1: c.861_862insT; p.P288SfsX22	No	Pathogenic
MDC1075	Lissencephaly	PAFAH1B1	NM_000430:c.(?_-451)_(32+1_33–1)del	No	Pathogenic
MDC1009	Lissencephaly	ARX	NM_139058: c.1034G>C; pR345P	No	Likely pathogenic
MDC1039	Lissencephaly	ARX	NM_139058:c.1427_1428delTCinsAA;p.F476X	No	Pathogenic

^1^ Parrini, E., et al. (2006). Periventricular heterotopia: phenotypic heterogeneity and correlation with Filamin A mutations.Brain 129(Pt 7): 1892–1906.

^2^ Reinstein E, Frentz S, Morgan T, García-Miñaúr S, Leventer RJ, McGillivray G et al. (2013). Vascular and connective tissue anomalies associated with X-linked periventricular heterotopia due to mutations in Filamin A. Eur J Hum Genet 21, 494 and Solé (2009) J Neurol Neurosurg Psychiatry 80, 1394

^3^ des Portes V1, Francis F, Pinard JM, Desguerre I, Moutard ML, Snoeck I et al.(1998) Doublecortin is the major gene causing X-linked subcortical laminar heterotopia (SCLH).Hum Mol Genet. Jul;7(7):1063–70

**Table 3:** Details of somatic mutations.

PARTICIPANT NUMBER	CORTICAL MALFORMATION	GENOTYPE							
		GENE	MUTATION	TYPE	READ- DEPTH	ALTERNATE ALLELE NUMBER OF READS	ALTERNATE-ALLELE READ FREQUENCY	ESTIMATED MUTANT CELL FREQUENCY	PREVIOUSLY REPORTED
MDC1092	SBH	DCX	NM_178152.1:c.235_491delinsTG	Deletion	2727*	1691	62%	62%	No
MDC1093	SBH	DCX	NM_178152.1: c.752C>T; p.A251V	Missense	6222	823	12.4%	24.8%	Yes ^1^
MDC1034	SBH	DCX	NM_178152.1: c.176G>A; p.R59H	Missense	4004	1911	48.7%	48,70%	Yes ^2^
MDC1070	SBH	PAFAH1B1	NM_000430: c.628G>C;p.A210P	Missense	4144	794	14.9%	29.8%	No

^1^ Sakamoto M1, Ono J, Okada S, Nakamura Y, Kurahashi H.(2000). Genetic alteration of the DCX gene in Japanese patients with subcortical laminar heterotopia or isolated lissencephaly sequence. J Hum Genet.45(3):167–70.

^2^ Gleeson JG1, Minnerath SR, Fox JW, Allen KM, Luo RF, Hong SE, (1999). Characterization of mutations in the gene doublecortin in patients with double cortex syndrome. Ann Neurol. Feb;45(2):146–53 and Matsumoto N, Leventer RJ, Kuc JA, Mewborn SK, Dudlicek LL, et al. (2001) Mutation analysis of the DCX gene and genotype/phenotype correlation in subcortical band heterotopia. Eur J Hum Genet 9: 5–12.

*Average coverage of the region.

### SBH

Seven patients with SBH were female (58%), thus representing a similar female/male ratio compared to other series [5]. All of them were sporadic cases, except MDC1045, a woman with SBH with a son affected by lissencephaly (SBH/LIS x-linked pedigree). The heterotopic band was bilateral and diffuse in all cases. A severe form with band thickness grade 3–4 (grade 3 = 8 to12 mm; grade 4 > 12 mm) was present in 50% of the patients and a milder form with band thickness grade 1–2 (grade 1 < 4 mm; grade 2 = 4 to 7 mm) in the other 50%.

We identified a pathogenic mutation in 7/12 patients. Remarkably, 4 mutations (57%) were somatic, including a PAFAH1B1 mutation in a girl with a p>a gradient (see clinical reports section bellow, MDC1070). The phenotypes in these 4 cases with a mosaic state were milder than expected for a germinal mutation: two males with DCX mutations (MDC1034 and MDC1092) and a woman with a PAFAH1B1 mutation (MDC1070), presenting with HBS instead of lissencephaly; and a woman with a somatic DCX mutation with a very thin predominantly anterior band of less than 4 mm without intellectual disability.

### PNH

Nineteen patients with PNH were included. Heterotopic nodules were bilateral (n = 18), or unilateral (n = 1). We found a *FLNA* mutation in 4 individuals, all of them with at least one clinical or radiological feature supportive of a *FLNA* mutation: MDC1002, typical PNH and vermis hypoplasia; MDC1005, typical PNH and cardiac (ventricular septal defect); MDC 1019, typical PNH and family history of recurrent spontaneous abortions; MDC 1020, male foetus stillbirth.

### Lissencephaly

We studied 7 patients with lissencephaly by means of MLPA and /or gene sequencing. One of them had frontal pachygyria (a >p gradient), and two had frontal pachygiria with posterior agyria (p >a gradient) A de novo *PAFAH1B1* deletion of exon 1 and was found in MDC1075, one of the patients with a p >a gradient. MLPA and PAFAH1B1 sequencing was normal in the rest. The other four patients had lissencephaly in association with Corpus Callosum agenesia and abnormal genitalia (XLAG). Regarding this last subgroup, two mutations in *ARX* were found in two cases.

### Genetic findings

#### Germline mutations

We successfully sequenced coding regions of *DCX*, *FLNA*, *PAFAH1B1* or *ARX* in 24 patients and found 8 different germline mutations in 9 patients: 3 in *FLNA*, 3 in *DCX* and 2 in *ARX*. Among them, 3 were nonsense (2 in *FLNA*, 1 in *ARX*), 4 were missense (1 in *FLNA*, 2 in *DCX*, 1 in *ARX*), whereas one was a 1 bp insertion that results in a frameshift of *DCX* translation that predicts the premature introduction of a stop signal. All variants detected by NGS were successfully validated by Sanger. All of the identified variants can be classified as pathogenic or likely pathogenic. Three of them were previously reported in individuals with a similar phenotype, while the other five were considered to be novel (Table 2). From this last group, two were null alleles, meaning there was a point mutation that translated into a gain of a stop codon (ARX:p.F476X and DCX:p.P288SfsX22), and three were predicted to result in a nonconservative missense substitution of an amino acid residue (FLNA:p.G1387S, DCX:p.R102P and ARX: pR345P), being absent in population genomic databases. These three variants represent drastic changes in the amino acid sequence, from a small flexible amino acid as glycine to a polar one capable of doing sulfured bonds as cysteine in the FLNA gene, or from the positive charged arginine to the rigid proline in both DCX and ARX genes. The positions involved in these changes are conserved in the phylogeny and are predicted to be pathogenic by several prediction tools (S3 Table). Addiionally, ARX: pR345P is located in the *ARX* homeodomain a DNA binding domain strongly associated with XLAG phenotypes.

#### Copy number variants

Patients with lissencephaly or SBH, but negative for *DCX* mutations, were subsequently studied for copy number variants. Successful MLPA results were obtained for 9 out of 9 SBH/lissencephaly samples. A single copy loss of *PAFAH1B1* exon 1 and 2 was detected in one sample with lissencephaly. The MLPA probe sets around this region covered all exons of *PAFAH1B1* and the close genes *METTL16* and *H1C1* (17p13.3 probes). The upstream breakpoint was estimated to be localized between *H1C1* and *METTL16* genes and the downstream breakpoint to be between exon 2 and 3 of *PAFAH1B1*. MLPA performed in the parents showed two copies of exon 1 and 2, suggesting a de novo deletion.

#### Somatic mutations

We applied targeted high-coverage NGS to 12 patients. We obtained a mean coverage of about 4000x. Pathogenic or likely pathogenic variants were found in 4 subjects, including 2 variants (in *PAFAH1B1* and *DCX* genes) with an alternate-allele read frequency lower than 15% (12–15%) and 2 *DCX* variants with and alternate-allele read frequency higher than 40%. These latter two were identified in two males with SBH (MDC1034 and MDC1092). Since *DCX* is localized on the X chromosome, and both individuals had a normal karyotype (46 XY), we concluded that this *pseudo heterozygosity* represented a mosaic state. Further validation with Sanger, confirmed these findings. Noteworthy, one of them consisted of a novel intraexonic 257 bp deletion and 2 pb insertion of *DCX* (S1 Fig) maintaining the coding frame but severely truncating the protein sequence. Since the alternate-allele read frequency is near 50%, these postzygotic mutations must have probably occurred in early embryonic stages of subjects MDC1034 and MDC1092.

On the other hand, Sanger sequencing would not have been sensitive for those two variants having alternate-allele read frequencies of less than 15%: DCX:p.A251V, previously reported as pathogenic and PAFAH1B1: p.A210P, a novel mutation (Table 3). Thus, we validated them by means of subcloning assays, followed by sequencing of individual colonies (S3 Fig). The latter novel mutation (PAFAH1B1: p.A210P) comprises the substitution of a highly conserved alanine to a proline, a rigider amino acid. This change is predicted to be damaging by several pathogenicity predictors. In addition, we studied the stability of the protein using a modelled peptid after the 1VYH structure in PDB, and evaluated the free energy difference between the wild and mutated protein using FoldX, resulting in a highly destabilizing change (> 4,06 Kcal/mol).

All phenotypes caused by mosaic mutations were milder than expected for germline mutations. MDC1093 had a diffuse SBH without cognitive delay and MDC1070 was a female with a mosaic mutation in *PAFAH1B1* that led to SBH instead of lissencephaly. Interestingly, the SBH had a posterior predominance, as seen in *PAFAH1B1* associated lissencephaly (see clinical report below).

### Phenotype-genotype correlations. Clinical reports

Here, we present a further description of some illustrative cases.

#### Periventricular nodular heterotopia—FLNA missense mutation with wide phenotypic spectrum

**Fig 2:**
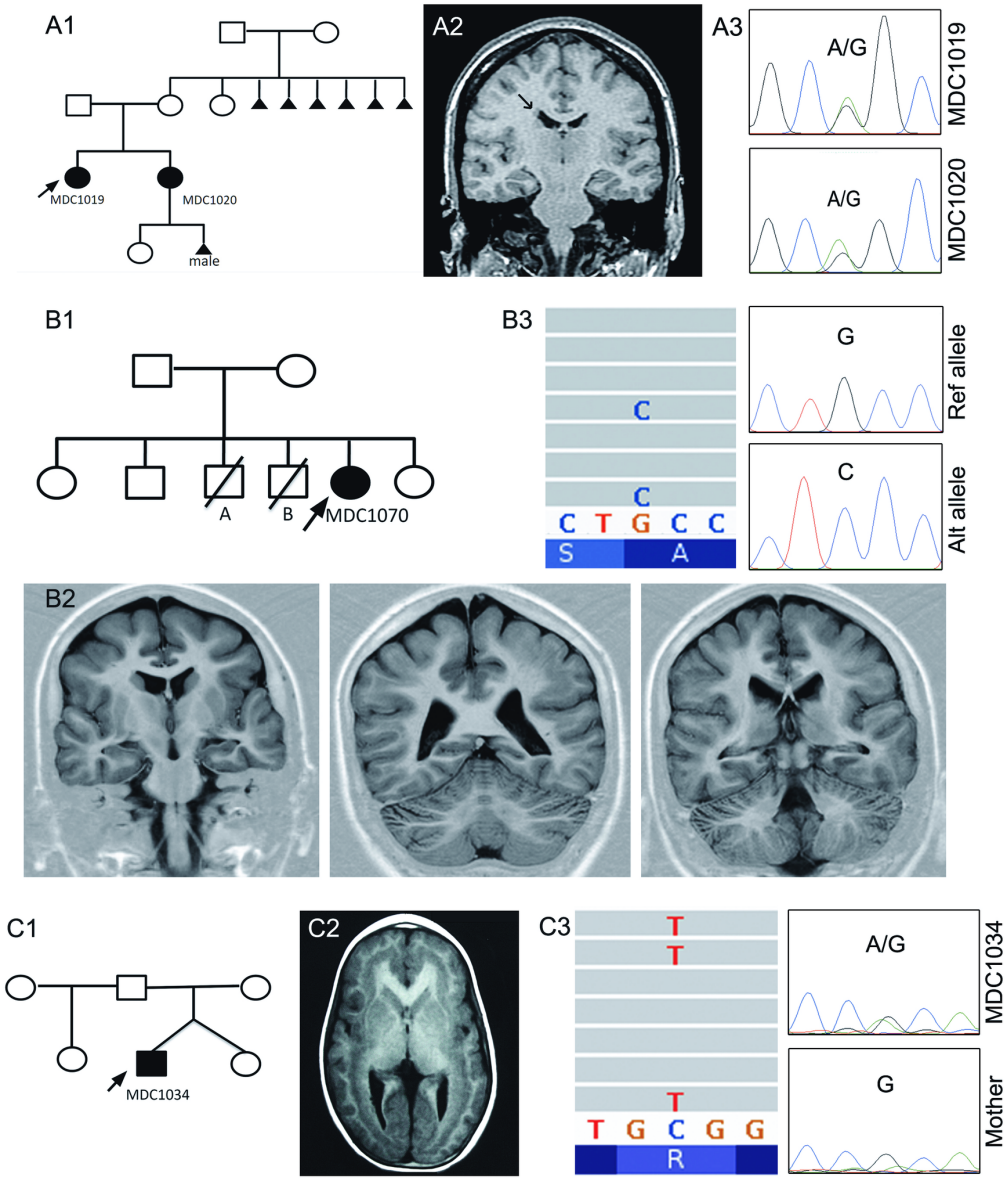
Pedigree structures, brain MRI and molecular findings for individuals MDC1019 (A1, A3), MDC1020 (A1, A2, A3), MDC1070 (B1, B2, B3) and MDC1034 (C1, C2, C3). A2. Coronal T1 MRI image shows isolated heterotopic nodule adjacent to the right lateral ventricle (arrow). B2. Inversion-Recovery Coronal MRI images show a posterior (P > A) band of subcortical heterotopia as well as simplified gyri and a thin layer of white matter between the cortex and band.C2. Coronal T1-WI shows a diffuse thick (>12mm) subcortical heterotopic band. A3. Sanger sequencing of *FLNA* gene showing the presence of both alleles in patients MDC1019 and 1020. B3. NGS (left) and Sanger sequencing after subcloning (right) of *PAFAH1B1* gene of patient 1070, showing the presence of the somatic mutation (alt allele) in both cases. C3. NGS (left) and Sanger (right) sequencing for *DCX* gene of patient 1034. The mutation is present at the X chromosome of the patient (male) but absent in the mother. Please consider that the Sanger sequencing was performed on the coding strand (reverse of reference).

MDC1019 is a 19-year-old woman with normal early development who presented focal seizures since she was 6 years old. The MRI revealed bilateral and diffuse nodular heterotopía. Although her sister, MDC1020, was totally asymptomatic regarding epilepsy and showed a normal neurological development, she reported the antecedent of one male foetus stillbirth (Fig 2-A1). MRI performed to MDC1020 revealed the presence of a single heterotopic nodule adjacent to the lateral ventricle (Fig 2-A2). Although MRI could not be performed to their mother to determine if she had nodular heterotopia, their grandmother had suffered multiple spontaneous abortions, a well-known consequence of *FLNA* mutations.

A novel missense mutation (c.4159G>A; p.G1387S) in the *FLNA* gene was found. Different tools predicted deleterious impact for this variant (SIFT-Polyphen- Mutation Taster) (Fig 2-A3). This mutation was later confirmed in her sister PC.

#### Agyria-pachygyria spectrum: Posterior SBH in a girl mosaic for a PAFAH1B1 mutation

MDC1070 was a female born at term. Her mother was asymptomatic, as well as two of her siblings. Conversely, two other brothers were dead before the first 2 months of life (Fig 2-B1). MDC1070 presented with development delay and during childhood, she developed drug-resistant epilepsy.

We decided to perform *DCX* sequencing first since 88% of females with SBH carry a *DCX* mutation. However, we were not able to find any *DCX* mutation by Sanger sequencing. *DCX* deletions and duplications were also discarded by MLPA. Subsequently, we did high coverage NGS searching for somatic mutations. Considering the posterior gradient of the cortical malformation (Fig 2-B2) we included also *PAFAH1B1* in the analysis. Again, no mutation in *DCX* could be identified but *PAFAH1B1* sequencing showed a mosaic missense mutation in *PAFAH1B1* with an alternate allele frequency of 14.9% (Fig 2-B3)

#### SBH in males—Somatic DCX mutation

MDC1034 is a male with no relevant family history that presented drug resistant epilepsy and developmental delay. His sister, a dizygotic twin, was asymptomatic (Fig 2-C1). No other family members were affected. MRI, in this case, showed a diffuse band of subcortical heterotopia (thickness> 12 mm) with a *a = p gradient* (Fig 2-C2).

*DCX* sequencing by NGS (genomic DNA extracted from peripheral blood leukocytes) revealed the mutation c.176G> A; p.R59H with an alternate-allele read frequency of 48.7% (Fig 2-C3). Given that *DCX* gene is located on the X chromosome and that our patient had a normal karyotype (46XY), this results can be considered a mosaic state in pseudo heterozygosity. These findings were subsequently confirmed by Sanger sequencing and subcloning followed by sequencing of individual colonies.

Finally, to explore the existence of mosaicism in other tissues, we studied genomic DNA extracted from epithelial cells obtained by buccal smear. Again, the c.176G> A substitution was revealed [15]. Mutational analysis in his mother showed a wild-type genotype.

## Discussion

We presented clinical, neuroimaging and genetic data of a large cohort of NMD patients followed in an academic neurogenetic tertiary center in Argentina. Our results expand the mutation spectrum causing this group of disorders and give new insights about the evolving role of somatic mutations in neurological diseases, especially for diagnostic purposes. A phenotype-driven comprehensive strategy looking for germline and mosaic sequence variants could identify a disease-causing defect in 36% of our patients. These results highlight the genetic heterogeneity of NMD, along with their complex phenotypes, that often lead to difficult diagnostic scenarios.

Most of the previous reports were retrospective, included selected homogeneous cohorts and, many times, they were restricted to the analysis of a single gene [5, 17]. Among them, the diagnostic yield was variable and dependent on selected phenotypes [18]. As an example, the chance of finding a mutation in *FLNA* in PNH patients could be as low as 4% within subjects with a non-classical phenotype, whereas it approximates 100% when only familial cases with X-linked PNH are considered [19]. On the contrary, we aimed to perform a prospective and comprehensive mutational analysis including typical and atypical phenotypes in our cohort. It is plausible that this heterogeneity resulted in a lower number of positive cases, but we believe that it comes closer to a *real-life* scenario. To our knowledge, this is the largest NMD series in Latin America reporting a comprehensive mutation spectrum.

In addition, our approach investigated not only germline variants but also mosaicism. Mosaic mutations might play a role in the phenotypic heterogeneity observed in NMD. Recent works demonstrated that early embryonic cell-doubling events contribute asymmetrically to adult tissues [20] because of stochastic allocation of embryonic cells in the inner cell mass. Thus, it is plausible that somatic mutations involving NMD genes may lead to diverse degrees of adult tissue affectation (and phenotypic severity) even when they occur during early embryonic stages. Germline *DCX* mutations in males and *PAFAH1B1* in either sex result in lissencephaly. However, when mutations are mosaic a milder severity could be expected, such as the phenotypes observed in patients MDC1070 and MDC1034 [21].

Also, somatic mosaicism could be behind the *missing genetic* cause of several NMD cases. *De novo* mutations are frequently found in this group of malformations [5]. Furthermore, early embryonic mutation rate seems to be similar to, or even slightly higher than, germline mutational rates [20]. Thus, we hypothesized that postzygotic mosaic mutations, could be detected more often in sporadic NMD, if they were properly investigated. Supporting this, a substantial fraction (29%) of the 14 mutations found in our study were postzygotic mosaic mutations. Jamuar et al performed the most extensive investigation of somatic mutations in brain cortical malformations in 2014.[4] In their study, they investigated 158 persons with brain malformations and found a causal mutation in 27, 30% of which were somatic. Thus, our findings are in concordance with those obtained by Jamuar et al. Furthermore, in their series as well as in ours, mosaic mutations were mainly in individuals with Double-Cortex syndrome (SBH),

Also, Zillhart et al.[22] have recently communicated that parental germline mosaicism accounts for about 15% of recurrent forms of MCD.

Overall, this evidence suggest that somatic mosaicism is not uncommon in MCD, specially in SBH, and call for a reappraisal of the most appropriate diagnostic strategies in clinical practice, In this aspect, our results, in addition to those of Jamuar et al. support the utility of targeted high coverage NGS for this purpose.

Considering our results, we propose a candidate gene targeted strategy only for patients presenting with a classical phenotype (eg. classical PNH in females), which in our opinion, should be implemented through NGS. This technique is more cost-effective for large genes such as *FLNA* [23] and it is more sensitive to detect mosaic mutations [24] In atypical cases, a more comprehensive approach that includes the scrutiny of multiple genes by high-coverage NGS based panels and structural variants detection by means of MLPA or microarray would be warranted [25]. Although, we did not perform whole exome (WES) or genome sequencing (WGS) in our work, they would certainly have been the next step in negative cases [26].

Delineating the several molecular mechanisms that disrupt neuronal migration might facilitate therapeutics discoveries while providing an improved understanding of normal brain development. However, since most of the neuronal migration disorders remain still genetically unexplained, we must remember that the molecular diagnosis of these malformations is a real challenge even in this current and promising genomic era.

## Supporting information

S1 Table Phenotypic characteristics of the NMD cohort. (XLSX)

S2 Table Somatic mutations. NGS data, in silico analysis of pathogenicity and population frequency is shown for pathogenic or likely pathogenic variants.
(XLSX)

S3 Table Germinal novel mutations. In silico analysis of pathogenicity.
(XLSX)

S1 Fig Somatic mutations: Intraexonic deletion of patient MDC 1092. A Targeted high coverage NGS. Exon 2 of patient MDC 1092 compared to normal exon 2 of patient MDC 1093. In patient 1092 the average coverage is significantly lower in the deleted fragment. B.Sanger sequencing after subcloning.
(XLSX)

S2 Fig Sanger electropherogram confirming the NM_139058:c.1427_1428delTCinsAA; p.F476X in patient 1039 (male). As ARX is located in the X chromosome only one allele is represented.
(XLSX)

S3 Fig Somatic mutations: Sanger sequencing after subcloning. Individuals MDC 1070 and MDC 1093.
(XLSX)

## References

[1] Guerrini R, Parrini E. Neuronal migration disorders. Neurobiol Dis. 2010;38(2):154–66. Epub 2009/02/28. doi: 10.1016/j.nbd.2009.02.008 . 19245832 10.1016/j.nbd.2009.02.008

[2] Manzini MC, Walsh CA. What disorders of cortical development tell us about the cortex: one plus one does not always make two. Current opinion in genetics & development. 2011;21(3):333–9. Epub 2011/02/04. doi: 10.1016/j.gde.2011.01.006 . 21288712 10.1016/j.gde.2011.01.006 PMC3139684

[3] Parrini E, Conti V, Dobyns WB, Guerrini R. Genetic Basis of Brain Malformations. Molecular syndromology. 2016;7(4):220–33. Epub 2016/10/27. doi: 10.1159/000448639 . 27781032 10.1159/000448639 PMC5073505

[4] Jamuar SS, Lam AT, Kircher M, D'Gama AM, Wang J, Barry BJ, et al Somatic mutations in cerebral cortical malformations. The New England journal of medicine. 2014;371(8):733–43. Epub 2014/08/21. doi: 10.1056/NEJMoa1314432 . 25140959 10.1056/NEJMoa1314432 PMC4274952

[5] Bahi-Buisson N, Souville I, Fourniol FJ, Toussaint A, Moores CA, Houdusse A, et al New insights into genotype-phenotype correlations for the doublecortin-related lissencephaly spectrum. Brain. 2013;136(Pt 1):223–44. Epub 2013/02/01. doi: 10.1093/brain/aws323 . 23365099 10.1093/brain/aws323 PMC3562079

[6] Mandelstam SA, Leventer RJ, Sandow A, McGillivray G, van Kogelenberg M, Guerrini R, et al Bilateral posterior periventricular nodular heterotopia: a recognizable cortical malformation with a spectrum of associated brain abnormalities. AJNR American journal of neuroradiology. 2013;34(2):432–8. Epub 2013/01/26. doi: 10.3174/ajnr.A3427 . 23348762 10.3174/ajnr.A3427 PMC7965117

[7] Gonzalez G, Vedolin L, Barry B, Poduri A, Walsh C, Barkovich AJ. Location of periventricular nodular heterotopia is related to the malformation phenotype on MRI. AJNR American journal of neuroradiology. 2013;34(4):877–83. Epub 2012/10/16. doi: 10.3174/ajnr.A3312 . 23064591 10.3174/ajnr.A3312 PMC3951137

[8] Kauffman MA, Gonzlez-Moron D, Consalvo D, Westergaard G, Vazquez M, Mancini E, et al Diagnosis of mitochondrial disorders applying massive pyrosequencing. Molecular biology reports. 2012;39(6):6655–60. Epub 2012/02/04. doi: 10.1007/s11033-012-1471-9 . 22302390 10.1007/s11033-012-1471-9

[9] de Wit MC, de Coo IF, Lequin MH, Halley DJ, Roos-Hesselink JW, Mancini GM. Combined cardiological and neurological abnormalities due to filamin A gene mutation. Clinical research in cardiology: official journal of the German Cardiac Society. 2011;100(1):45–50. Epub 2010/08/24. doi: 10.1007/s00392-010-0206-y . 20730588 10.1007/s00392-010-0206-y PMC3022162

[10] DePristo MA, Banks E, Poplin R, Garimella KV, Maguire JR, Hartl C, et al A framework for variation discovery and genotyping using next-generation DNA sequencing data. Nat Genet. 2011;43(5):491–8. Epub 2011/04/12. doi: 10.1038/ng.806 . 21478889 10.1038/ng.806 PMC3083463

[11] Van der Auwera GA, Carneiro MO, Hartl C, Poplin R, Del Angel G, Levy-Moonshine A, et al From FastQ data to high confidence variant calls: the Genome Analysis Toolkit best practices pipeline. Current protocols in bioinformatics. 2013;43:11 0 1–33. Epub 2014/11/29. doi: 10.1002/0471250953.bi1110s43 . 25431634 10.1002/0471250953.bi1110s43 PMC4243306

[12] McKenna A, Hanna M, Banks E, Sivachenko A, Cibulskis K, Kernytsky A, et al The Genome Analysis Toolkit: a MapReduce framework for analyzing next-generation DNA sequencing data. Genome Res. 2010;20(9):1297–303. Epub 2010/07/21. doi: 10.1101/gr.107524.110 . 20644199 10.1101/gr.107524.110 PMC2928508

[13] Rimmer A, Phan H, Mathieson I, Iqbal Z, Twigg SR, Consortium WGS, et al Integrating mapping-, assembly- and haplotype-based approaches for calling variants in clinical sequencing applications. Nat Genet. 2014;46(8):912–8. Epub 2014/07/16. doi: 10.1038/ng.3036 . 25017105 10.1038/ng.3036 PMC4753679

[14] Rausch T, Zichner T, Schlattl A, Stutz AM, Benes V, Korbel JO. DELLY: structural variant discovery by integrated paired-end and split-read analysis. Bioinformatics. 2012;28(18):i333–i9. Epub 2012/09/11. doi: 10.1093/bioinformatics/bts378 . 22962449 10.1093/bioinformatics/bts378 PMC3436805

[15] Cingolani P, Platts A, Wang le L, Coon M, Nguyen T, Wang L, et al A program for annotating and predicting the effects of single nucleotide polymorphisms, SnpEff: SNPs in the genome of Drosophila melanogaster strain w1118; iso-2; iso-3. Fly. 2012;6(2):80–92. Epub 2012/06/26. doi: 10.4161/fly.19695 . 22728672 10.4161/fly.19695 PMC3679285

[16] Richards S, Aziz N, Bale S, Bick D, Das S, Gastier-Foster J, et al Standards and guidelines for the interpretation of sequence variants: a joint consensus recommendation of the American College of Medical Genetics and Genomics and the Association for Molecular Pathology. Genetics in medicine: official journal of the American College of Medical Genetics. 2015;17(5):405–24. Epub 2015/03/06. doi: 10.1038/gim.2015.30 . 25741868 10.1038/gim.2015.30 PMC4544753

[17] Matsumoto N, Leventer RJ, Kuc JA, Mewborn SK, Dudlicek LL, Ramocki MB, et al Mutation analysis of the DCX gene and genotype/phenotype correlation in subcortical band heterotopia. European journal of human genetics: EJHG. 2001;9(1):5–12. Epub 2001/02/15. doi: 10.1038/sj.ejhg.5200548 . 11175293 10.1038/sj.ejhg.5200548

[18] D'Agostino MD, Bernasconi A, Das S, Bastos A, Valerio RM, Palmini A, et al Subcortical band heterotopia (SBH) in males: clinical, imaging and genetic findings in comparison with females. Brain. 2002;125(Pt 11):2507–22. Epub 2002/10/23. . 12390976 10.1093/brain/awf248

[19] Parrini E, Ramazzotti A, Dobyns WB, Mei D, Moro F, Veggiotti P, et al Periventricular heterotopia: phenotypic heterogeneity and correlation with Filamin A mutations. Brain. 2006;129(Pt 7):1892–906. Epub 2006/05/11. doi: 10.1093/brain/awl125 . 16684786 10.1093/brain/awl125

[20] Ju YS, Martincorena I, Gerstung M, Petljak M, Alexandrov LB, Rahbari R, et al Somatic mutations reveal asymmetric cellular dynamics in the early human embryo. Nature. 2017;543(7647):714–8. Epub 2017/03/23. doi: 10.1038/nature21703 . 28329761 10.1038/nature21703 PMC6169740

[21] Quelin C, Saillour Y, Souville I, Poirier K, N'Guyen-Morel MA, Vercueil L, et al Mosaic DCX deletion causes subcortical band heterotopia in males. Neurogenetics. 2012;13(4):367–73. Epub 2012/07/27. doi: 10.1007/s10048-012-0339-4 . 22833188 10.1007/s10048-012-0339-4

[22] Zillhardt JL, Poirier K, Broix L, Lebrun N, Elmorjani A, Martinovic J, et al Mosaic parental germline mutations causing recurrent forms of malformations of cortical development. European journal of human genetics: EJHG. 2016;24(4):611–4. Epub 2015/09/24. doi: 10.1038/ejhg.2015.192 . 26395554 10.1038/ejhg.2015.192 PMC4929884

[23] van Nimwegen KJ, van Soest RA, Veltman JA, Nelen MR, van der Wilt GJ, Vissers LE, et al Is the $1000 Genome as Near as We Think? A Cost Analysis of Next-Generation Sequencing. Clinical chemistry. 2016;62(11):1458–64. Epub 2016/10/30. doi: 10.1373/clinchem.2016.258632 . 27630156 10.1373/clinchem.2016.258632

[24] Qin L, Wang J, Tian X, Yu H, Truong C, Mitchell JJ, et al Detection and Quantification of Mosaic Mutations in Disease Genes by Next-Generation Sequencing. The Journal of molecular diagnostics: JMD. 2016;18(3):446–53. Epub 2016/03/06. doi: 10.1016/j.jmoldx.2016.01.002 . 26944031 10.1016/j.jmoldx.2016.01.002

[25] Jamuar SS, Walsh CA. Genomic variants and variations in malformations of cortical development. Pediatric clinics of North America. 2015;62(3):571–85. Epub 2015/05/30. doi: 10.1016/j.pcl.2015.03.002 . 26022163 10.1016/j.pcl.2015.03.002 PMC4449454

[26] Hertecant J, Komara M, Nagi A, Suleiman J, Al-Gazali L, Ali BR. A novel de novo mutation in DYNC1H1 gene underlying malformation of cortical development and cataract. Meta gene. 2016;9:124–7. Epub 2016/06/23. doi: 10.1016/j.mgene.2016.05.004 . 27331017 10.1016/j.mgene.2016.05.004 PMC4908276

